# A successfully resected case of left trisectionectomy with arterio-portal combined resection for advanced cholangiocarcinoma

**DOI:** 10.1016/j.ijscr.2018.10.036

**Published:** 2018-10-25

**Authors:** Atsushi Nanashima, Naoya Imamura, Masahide Hiyoshi, Koichi Yano, Takeomi Hamada, Teru Chiyotanda, Kenzo Nagatomo, Rouko Hamada, Hiroshi Ito

**Affiliations:** a Division of Hepato-Biliary-Pancreas and Digestive Surgery in the Department of Surgery, University of Miyazaki, Faculty of Medicine, Kiyotake 5200, Miyazaki, 889-1692, Japan; b Division of Plastic and Reconstructive Surgery in the Department of Surgery, University of Miyazaki, Faculty of Medicine, Kiyotake 5200, Miyazaki, 889-1692, Japan

**Keywords:** Left trisectionectomy, Combined vascular resection, Careful managements

## Abstract

**Introduction:**

The present case report demonstrated the successfully radical operation (R0) for the highly advanced cholangiocarcinoma involving hilar hepatic arteries and portal vein, The careful preoperative diagnosis to define the adequate resection area and the expert operation was achieved without postoperative severe complications.

**Presentation of case:**

A 55-year-old male was admitted to our hospital with obstructive jaundice, and the perihilar cholangiocarcinoma (PC) was found. At the time of finding PC, enhanced computed tomography showed the widely extension and involved the surrounding right hepatic artery (RHA) and bilateral portal veins (PV). According to extension of PC, left trisectionectomy combined resection of RHA and PV trunk was scheduled. By supporting plastic surgeon’s procedure, the scheduled R0 operation could be achieved and the patient was discharged without any severe complication but delayed intrahepatic abscess formation. After abscess drainage, he could immediately recovered and tumor relapse was not observed for a couple of months. By carefully preoperative examination, a complicated operation was successfully completed.

**Discussion:**

The major hepatectomy with arterio-portal resections and anastomosis for advanced has been challenged at the high-volume center and the improvement of survival seemed to be obtained and, however, operative risk is still remained. This operation could be achieved by the expert surgeons under precise planning or management.

**Conclusion:**

The role of HBP surgeons is to challenge aggressive surgery even for patients with highly advanced local extension of PC.

## Highlights

• In case of cholangiocarcinoma invading hilar vessels, adequate simulations and expert skills are required to achieve R0 resection.

## 1 Introduction

R0 resection is the only curative treatment for perihilar cholangiocarcinoma (PC) [1,2], and aggressive surgery is necessary to ensure that the tumor is not exposed during the dissection procedure. In cases in which PC are surrounded by major blood vessels, appropriate diagnosis and surgical planning for combined resection, particularly regarding the location of the hepatic artery, are necessary to ensure that the treatment is curative and safe [3]. Proximal arterial or portal anastomoses, which require expert surgical skills, are sometimes needed, and preoperative imaging-based diagnosis and liver functional evaluations should be performed in such cases [4].

The present case report describes the successful treatment of extensive PC via R0 left trisectionectomy (LT) (resection of Couinaud’s segments 1, 2, 3, 4, 5, and 8) and careful preoperative and intraoperative management. This case has been reported in line with the SCARE criteria [5].

## 2 Presentation of case

**Fig. 1:**
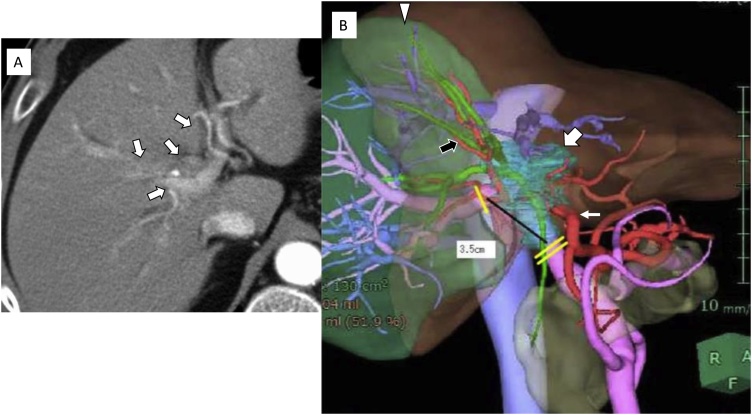
Contrast-enhanced CT showed an extensive PC, involving the middle and right hepatic arteries (white arrow); however, the posterior branch of the hepatic artery had not been invaded (a). Three-dimensional surgical planning images and fusion images composed of the planning images and cholangiographic images showed locally advanced PC, involving bile ducts, hepatic arteries, and the portal vein (the trunk and bilateral branches) (the blue area indicated by the thick white arrow) (b). The tumor had also invaded the anterior sectional bile duct as well as the anterior sectional hepatic artery and portal vein (black arrow), but the posterior equivalents of these structures had not been affected by the tumor. The proper hepatic artery had not been invaded by the tumor (thin white arrow). The volume of the future remnant liver (green area) was predicted to be 48% of the original liver volume.

A 55-year-old male was admitted to our hospital with obstructive jaundice. Imaging revealed a locally advanced PC, and endoscopic bile duct drainage and precise determination of the extent to which the tumor had invaded the intrahepatic bile ducts were performed. Contrast-enhanced abdominal computed tomography (CT) combined with three-dimensional imaging and cholangiography showed a perihilar mass lesion together with extensive stenosis of the intra- and extra-hepatic bile ducts (Fig. 1a and b). The mass was definitively diagnosed as a type IV PC, according to the Bismuth-Corlette classification of hilar cholangiocarcinoma. The tumor extended into the left hepatic duct and anterior sectional bile duct, and it had also invaded the adjacent right hepatic artery, and the left and anterior sectional branches of the portal vein. Endoscopic biopsies of the stenotic lesion and the non-stenotic bile ducts at the confluence of the posterior sectional bile duct branches (as a negative biopsy) were performed. Although invasive adenocarcinoma was diagnosed, no cancer infiltration was observed in the epithelium at the confluence of the posterior sectional bile ducts. As no distant or lymph node metastasis was detected, and the patient had a sufficient liver functional reserve for LT, we scheduled R0 resection accompanied by arterioportal resection and anastomosis. During the surgical simulation, it was determined that an arterial anastomosis should be performed unless the tumor had invaded the tissues surrounding the remnant posterior branch of the hepatic artery. In addition, it was decided that if R0 resection were not possible, then the scheduled LT would be abandoned.

**Fig. 2:**
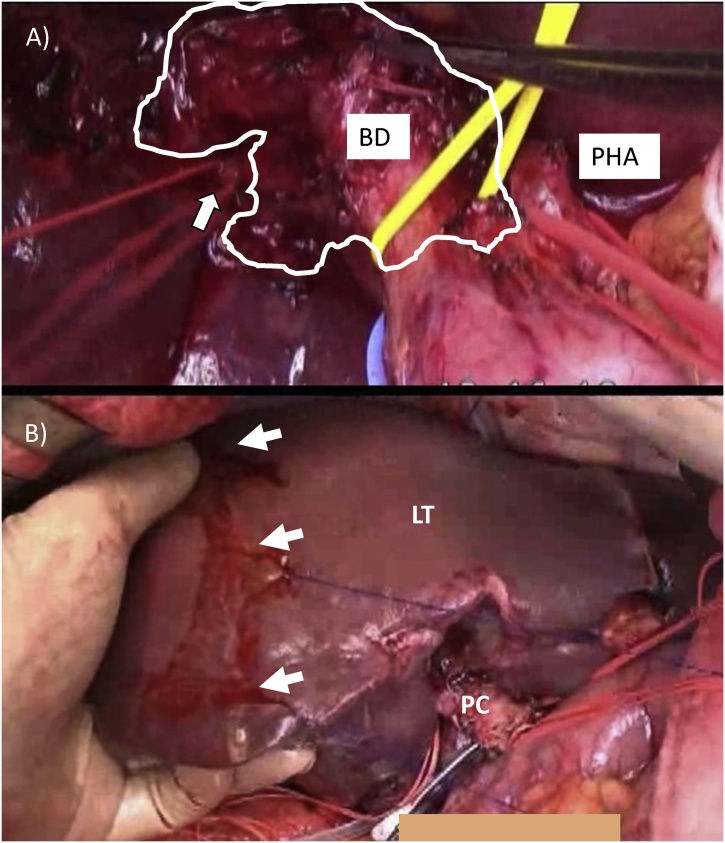
The encircled area shows the tumor mass (A). The arrow indicates the posterior hepatic artery (BD = bile duct, PHA = proper hepatic artery). The border between the posterior and anterior sectors of the liver is indicated by the arrow (PC = perihilar cholangiocarcinoma, LT = left trisectionectomy) (B).

**Fig. 3:**
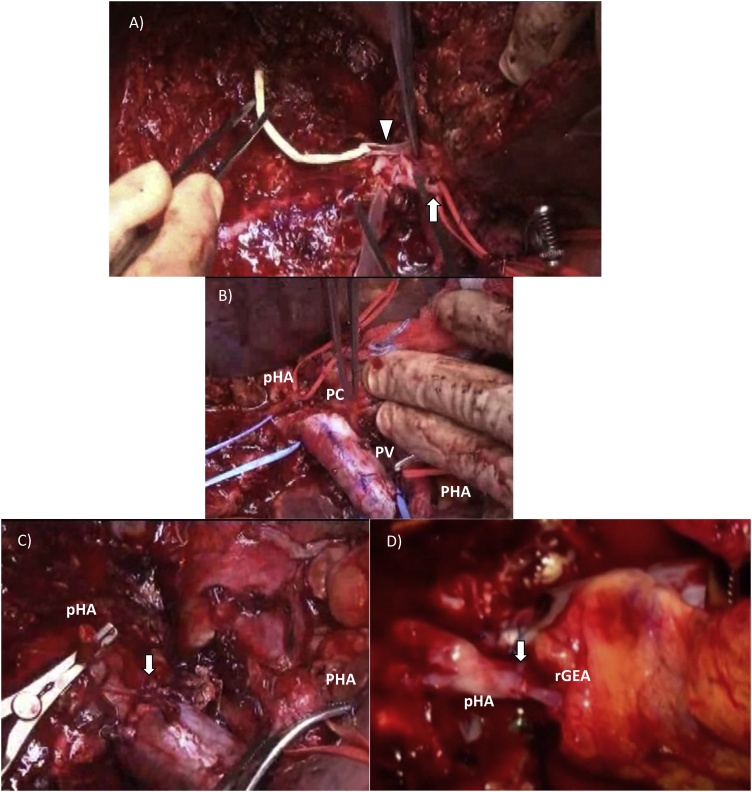
Severe adhesion between the hilar bile duct and right hepatic artery was observed (arrowhead) (A), and the resected specimen showed that the PC was only connected to the portal vein (PV) and right hepatic artery (pHA = posterior branch of the hepatic artery, PHA = proper hepatic artery) (B). Anastomosis of the portal vein (PV) was performed (arrow) (C). An arterial anastomosis was conducted under microscopy (D) (rGEA = right gastro-epiploic artery).

No peritoneal dissemination, liver metastasis, or distant lymph node metastasis was seen after laparotomy. Although a hard and fixed PC lesion was present (Fig. 2A), we attempted to sample some swollen lymph nodes and the tissues surrounding the posterior hepatic artery. No cancer cells were observed in any of the tissue samples, and therefore, we decided to proceed with the scheduled operation. The distal bile duct was transected at the level of the upper limit of the pancreatic head and was free from cancer. After transecting the left and middle hepatic arteries, a clear demarcation between the ischemic and well perfused tissue was seen (Fig. 2B). Hepatic parenchymal transection along the right hepatic vein was performed, and the left and middle hepatic veins and the posterior hepatic duct were transected (Fig. 3A). The right hepatic artery and portal vein were transected along the scheduled incision line (yellow lines in Fig. 1B) (Fig. 3B). A macroscopic examination of the resected specimen indicated that an R0 resection had been achieved without exposing the tumor. Next, a portal anastomosis was created between the posterior branch of the portal trunk, even though the orifices of these blood vessels differed markedly in size (Fig. 3C). After confirming the presence of good portal flow, an arterial anastomosis between a tiny branch of the portal vein and a branch of the right gastro-epiploic artery was created by a plastic surgeon under microscopy (Fig. 3D). Locoregional lymph node dissection was added. Hepaticojejunostomy with Roux-en Y jejuno-jejunostomy were also performed. The total operating time was 804 min (including 120 min for the arterial anastomosis), and 2430 ml of intraoperative blood loss occurred, which necessitated the transfusion of four units of red blood cells.

**Fig. 4:**
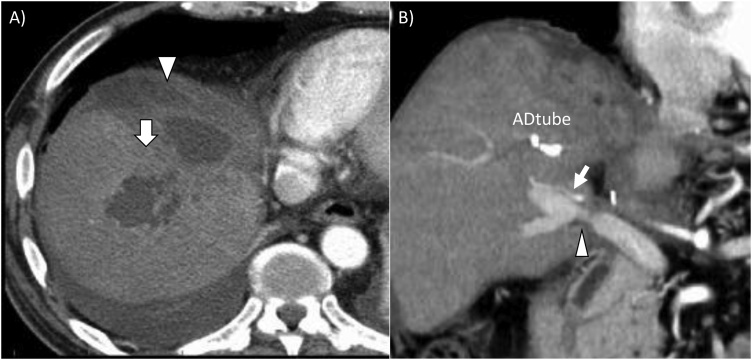
A delayed liver abscess developed in segment 7 (arrow), which caused the accumulation of perihepatic free fluid (arrowhead) (A). After intra-abscess drainage via percutaneous puncture (using an abdominal drainage tube), the abscess was relieved, and intra-hepatic arterial (arrow) and portal flow (arrowhead) were maintained (B).

A microscopic examination revealed poorly differentiated adenocarcinoma with arterial and portal invasion, but the tumor did not extend to the dissected surface. No lymph node metastasis was found. It was histologically confirmed that an R0 resection had been achieved. The patient’s postoperative course was uneventful, and no severe liver damage was seen in the remnant liver. Therefore, the patient was discharged on day 24. However, after being discharged the patient developed an intra-hepatic abscess in segment 7, and perihepatic free fluid started to accumulate (Fig. 4A and B), which was treated with drainage on day 91. On day 35 after the drainage procedure, the patient was discharged from hospital. At 6 postoperative months, the tumor had not recurred.

## 3 Discussion

Recently, major hepatectomy combined with vascular resection has been performed for PC after adequate preoperative evaluations of the functional liver reserve [3,[6], [7], [8]]. However, type IV PC, according to the Bismuth-Corlette classification, can be radically resected [9]. As it can be performed safely, and the prognosis of surgically treated PC patients is better than that of PC patients that do not undergo resection, LT with arterial or portal resection tends to be performed for PC at Japanese high-volume centers [3,[6], [7], [8], [9], [10]]. However, this procedure is considered to be associated with greater surgical risk than hemi-hepatectomy [11]. Additional adverse complications after such an operation can lead to systemic damage or lethal conditions, and therefore, careful selection of the optimal surgical procedure by expert hepatobiliary surgeons is necessary in cases of PC. We have performed LT and combined resection of the artery and portal vein in a few cases. In the present case, we read previous reports and fully checked the pitfalls of the abovementioned procedure during the preoperative period [3,[6], [7], [8], [9], [10]]. The tumor was relatively large, and tumor infiltration, as indicated by soft-tissue density, into the region around the posterior hepatic artery and the stump of the posterior branch of the bile duct was suspected based on preoperative CT. However, it was not possible to definitively determine the full extent of the tumor. On the other hand, we considered that aggressive treatment was necessary because the patient was relatively young. If cancer cells had been detected in the dissected tissue around the resection line during the operation or massive lymph node metastases had been observed, we would have discontinued the radical operation.

With respect to the intraoperative findings and procedures, we first performed lymphadenectomy from the common hepatic artery lesion to the hepatic hilum, and then extended it along the relevant arteries to detect distant lymph node metastases. The dissected soft tissue around the dissection line was hard, but no cancer cells were observed, and furthermore, no lymph node metastases were detected during intraoperative pathological examinations. Eventually, a histological examination showed that there were no lymph node metastases. At present, combined resection and anastomosis of the hilar blood vessels tends to be performed after complete parenchymal transection of the liver in cases of advanced PC [4,12]. During warm ischemia of the remnant liver, it is important to be aware of the re-perfusion time. Warm ischemia can be permitted for around 30 min. In the present case, the difference between the orifice sizes of the vessels to be anastomosed was quite large, and so careful anastomosis was necessary. The creation of arterial anastomoses should be entrusted to plastic or vascular surgeons and should be performed under microscopy [13]. The author reported that microsurgery had great benefits in a case in which it was used to create an arterial anastomosis during hepato-pancreato-biliary surgery [14]. Although the plastic surgeon in the current case took a long time to create the anastomosis, it is possible that such procedures could be performed quicker as plastic surgeons gain experience of abdominal surgery.

Fortunately, in the present case an R0 resection was achieved, and the patient’s postoperative course was good. However, delayed intra-hepatic abscess formation [15] occurred after 80 days. Despite this, the patient’s vascular flow and liver function were well maintained. The possible reasons for the abscess formation were considered to be as follows: 1) partial arterial ischemia, 2) peripheral cholangitis due to reflux via the hepaticojejunostomy, and 3) remnants of the transected Glissonian branches of segment 8 were present beyond the right hepatic vein. Intra-abscess drainage was performed, but the subsequent CT did not provide any evidence of the cause of the abscess formation. As biliary leaks are common postoperative complications, hepatobiliary iminodiacetic acid or magnetic resonance cholangiography can be used to confirm the etiology of perihepatic free fluid accumulation or hepatic collection/abscess formation. It is necessary to manage patients who undergo complicated operations carefully after discharge, as delayed diagnosis or treatment of complications can lead to lethal sepsis. In the present case, adequate drainage and ascites control were employed, which resulted in the patient exhibiting a good course after discharge. If it had been found that the abscess formation was caused by occlusion of the hepatic artery, an arterioportal bypass operation to maintain the intrahepatic oxygen concentration would have been considered to rescue the situation [16].

## 4 Conclusion

We reported a case in which advanced PC was successfully treated using LT combined with arterioportal resection and anastomosis. An R0 resection and a good postoperative course were achieved; however, delayed intrahepatic abscess formation occurred. In such cases, precise determination of the extent of the tumor and evaluations of liver function should be performed preoperatively, and appropriate surgical simulations using three-dimensional imaging are important. Such efforts might contribute to expanding the surgical indications for hepatobiliary cancer and help to achieve safe R0 resection. Support by expert surgeons and physicians is necessary to achieve good patient outcomes in such cases.

## Conflicts of interest

No COI.

## Sources of funding

No funding.

## Ethical approval

Ethical permission for case report is obtained at our intuitional policy.

## Consent

Informed consent was obtained in this patient. Written informed consent was obtained from the patient for publication of this case report and accompanying images. A copy of the written consent is available for review by the Editor-in-Chief of this journal on request.

## Author contribution

All authors contributed the perioperative management and writing this paper. A.N., the first author, is a main operator and wrote this mainly.

A.N., N.I., Y.T., M.H., K.Y., T.H, T.C., K.N.and Y.F. contributed patient operation and perioperative management equally in this case report. A.N. was chairman and director of the department and a main operator. H.I. supported the arterial anastomosis during operation. All authors approved the final version of the manuscript to be submitted.

## Registration of research studies

N/A.

## Guarantor

Professor K.N, who is a cardiovascular surgeon, who is another chairman of our institute.

## Submission declaration

The authors declare that the work described has not been published previously, that it is not under consideration for publication elsewhere, that its publication has been approved by all authors and either tacitly or explicitly by the responsible authorities where the work was carried out, and that, if accepted, it will not be published elsewhere—including electronically in the same form in English or any other language—without the written consent of the copyright holder.

## Provenance and peer review

Not commissioned externally peer reviewed.
